# A test of the Archaic *Homo* Introgression Hypothesis for the Chiari malformation type I

**DOI:** 10.1093/emph/eoaf009

**Published:** 2025-06-27

**Authors:** Kimberly Plomp, Daniel Lewis, Laura Buck, Shafqat Bukhari, Todd Rae, Kanna Gnanalingham, Mark Collard

**Affiliations:** School of Archaeology, University of the Philippines Diliman, Quezon City, Philippines; School of Biological Sciences, University of Manchester, Manchester, UK; Department of Neurosurgery, Manchester Centre for Clinical Neurosciences, Salford Royal NHS Foundation Trust, Manchester Academic Health Science Centre, Manchester, UK; Research Centre for Evolutionary Anthropology and Palaeoecology, School of Biological and Environmental Sciences, Liverpool John Moores University, Liverpool, UK; Department of Neurosurgery, Manchester Centre for Clinical Neurosciences, Salford Royal NHS Foundation Trust, Manchester Academic Health Science Centre, Manchester, UK; School of Life Sciences, University of Sussex, Sussex House, Falmer Brighton, UK; School of Biological Sciences, University of Manchester, Manchester, UK; Department of Neurosurgery, Manchester Centre for Clinical Neurosciences, Salford Royal NHS Foundation Trust, Manchester Academic Health Science Centre, Manchester, UK; Laboratory of Human Evolutionary Studies, Department of Archaeology, Simon Fraser University, Burnaby, British Columbia, Canada

**Keywords:** cerebellar herniation, cerebellum, hybridization, fossil hominin, 3D shape analysis, geometric morphometrics, human evolution, evolutionary medicine

## Abstract

The Chiari malformation type I (CM-I) is a herniation of the cerebellum through the foramen magnum. Its proximate cause is accepted to be an unusually small occipital bone. However, its ultimate cause remains unclear. In 2013, Fernandes and colleagues hypothesized that individuals develop CM-I because some of their cranial development-coding genes derive from three extinct *Homo* species that have smaller basicrania than is typical for modern humans—*Homo erectus*, *Homo heidelbergensis*, and *Homo neanderthalensis*. Here, we report a study in which we used 3D data and Geometric Morphometrics to evaluate this hypothesis. We began by investigating whether CM-I is associated with significant differences in cranial shape in a sample of living humans. Subsequently, we compared the crania of living humans with and without CM-I to fossil crania assigned to *H. erectus*, *H. heidelbergenesis*, *H. neanderthalensis*, and *H. sapiens*. The study’s results were mixed. The first set of analyses identified significant shape differences between the crania of people with CM-I and the crania of unaffected people, which is in line with the hypothesis. In contrast, the second set of analyses did not support the hypothesis. They indicated that the crania of living humans with CM-I are only closer in shape to one of the extinct species, *H. neanderthalensis*. The other two extinct species were found to be closer in shape to living humans without CM-I. This is contrary to the main prediction of the hypothesis. Together, our results suggest the hypothesis should be narrowed to focus on introgressed genes from Neanderthals.

## INTRODUCTION

The Chiari malformation type I (CM-I) is a developmental neurological condition in which the lower part of the cerebellum protrudes through the foramen magnum into the cervical spinal canal. First described in the 19th century CE by the Austrian pathologist Hans Chiari [1, 2], CM-I is thought to be related to an underdevelopment of the occipital bone, which creates a posterior cranial fossa that is too small and shallow to adequately house the cerebellum [3–5]. The condition is usually said to affect around 1 in 1000 people, but recent imaging studies suggest that the prevalence may be markedly higher, possibly in excess of 1 in 100 [4–6]. CM-I can be asymptomatic, and if symptoms do occur, they can vary considerably depending on the size of the herniation. Symptoms range from occipital-region headaches and neck pain to the development of hydrocephalus, syringomyelia, and brainstem compression [5, 7–9].

While there is a general consensus that the proximate cause of CM-I is an unusually small occipital bone, the ultimate cause (i.e. the cause of the unusually small occiput) is still unclear. Over the years, a number of potential aetiological factors for the underdevelopment of the occipital bone associated with the malformation have been proposed. Hans Chiari [1, 2] thought it was a consequence of foetal hydrocephaly. Subsequently, other researchers have suggested that it may be related to craniosynostosis, platybasia, or excessive *in utero* exposure to vitamin A [10–14]. It is possible, perhaps even likely, that all these factors can result in a smaller occipital bone and therefore in CM-I. However, the relationship between CM-I and each potential factor is inconsistent [15], which implies there may be another reason why some people develop this condition.

A little over a decade ago, Fernandes *et al*. [16] put forward a novel ultimate-level hypothesis for CM-I. They suggested that it is a consequence of interbreeding between early *Homo sapiens* and ancient *Homo* species. Ancient DNA (aDNA) analyses have shown that during the Pleistocene, some *H. sapiens* individuals interbred with *Homo neanderthalensis*, Denisovans (an as-yet undiagnosed taxon closely related to Neanderthals), and potentially other extinct hominin species [17], and the legacy of these interbreeding events can be identified in the genomes of many living humans [17, 18]. Fernandes *et al*. [16] drew on these findings. They proposed that individuals with CM-I possess introgressed genes that influence cranial development in such a way that there ends up being a mismatch between the size and shape of the brain and the size and shape of the cranium, especially the basicranium. The genes in question, Fernandes *et al*. [16] argued, derive from three archaic *Homo* species—*Homo erectus*, *Homo heidelbergensis*, and *H. neanderthalensis*. Hereinafter, we will refer to this hypothesis as the ‘Archaic *Homo* Introgression Hypothesis’.

**Figure 1.**
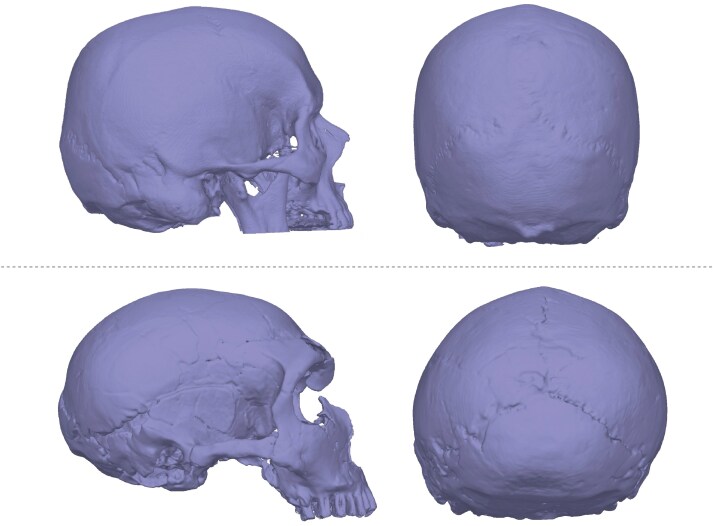
3D models of *Homo sapiens* (top two images) and *Homo neanderthalensis* (bottom two images) crania for visual comparison. The human model was created from DICOM files of an anonymised volunteer patient from the Manchester Centre for Clinical Neurosciences. The Neanderthal model is based on La Ferrassie 1 and was created by LB and TR.

The Archaic *Homo* Introgression Hypothesis is plausible when we consider the differences in cranial shapes between *H. sapiens* and the best known of the three archaic *Homo* species that Fernandes *et al*. [16] highlight in their hypothesis, *H. neanderthalensis* (Fig. 1). Typically, the modern human neurocranium is globular, with an upright forehead, the widest point high on the parietals, and a rounded occiput [19, 20]. In comparison, the Neanderthal neurocranium is lower and more elongated. The forehead is flatter, the widest point of the vault is lower on the parietals, and the occiput is more angled [21, 22]. These differences are thought to be driven largely by the greater size of the occipital and temporal lobes of the brain of our species compared to that of *H. neanderthalensis* [20, 23, 24].

Three recent studies provide indirect support for the Archaic *Homo* Introgression Hypothesis. Gregory *et al*. [22] analysed cranial traits of 221 healthy European adults in relation to the genes known to be derived from *H. neanderthalensis* and found that the amount of Neanderthal DNA in a person’s genome is positively correlated with the presence of Neanderthal-like cranial traits. Gunz *et al*. [23] analysed endocranial shape in relation to introgressed *H. neanderthalensis* DNA in thousands of living humans and found that the presence of certain Neanderthal alleles is associated with reduced globularity of the cranium. Kochiyama *et al*. [24] used endocranial reconstructions to compare brain shape in Neanderthals and modern humans. Although they did not include an analysis of introgressed genes, they did find that the greatest difference between the brains of the two species is in the cerebellum region. Specifically, they found that the modern human cerebellum is larger in volume and projects more inferiorly than that of the Neanderthal. This aligns with the pathogenesis of CM-I, as discussed earlier.

Here, we report a study designed to directly test the Archaic *Homo* Introgression Hypothesis. In the study, we used three-dimensional (3D) data and a suite of shape analysis techniques called geometric morphometrics (GM) to carry out two sets of analyses. In the first, we compared the crania of living people with and without CM-I. The goal of this set of analyses was to test the key assumption of the hypothesis, which is that CM-I is associated with significant differences in cranial shape, especially with respect to the basicranium. In the second set of analyses, we compared the crania of living people with and without CM-I to fossil crania assigned to *H. sapiens* and to the three extinct *Homo* species that Fernandes *et al*. [16] argued contributed genes to the modern human gene pool via interbreeding, i.e. *H. erectus*, *H. heidelbergenesis*, and *H. neanderthalensis*. The goal of this set of analyses was to test the main prediction of the hypothesis, which is that the crania of people with CM-I should be more similar to the crania of *H. erectus*, *H. heidelbergenesis*, and *H. neanderthalensis*, than the crania of people without CM-I.

## METHODS

We included data from 103 living humans in the study. All these individuals were adults at the time of data collection and had undergone thin-slice volumetric cranial CT scanning at the Manchester Centre for Clinical Neurosciences, UK. Ethics approval for the study was provided by the NHS Health Research Authority (NRES committee South Central Hampshire A 19/SC/0341) and all living participants provided informed consent for analysis of their data. Forty-six of the living individuals had CM-I. These individuals had undergone CT scanning as part of their diagnostic and surgical workup for CM-I. Patients with tonsillar ectopia less than 5 mm below the foramen magnum and other Chiari malformation types (types II, III, and IV) related to defective neurulation and neural tube closure during embryogenesis were excluded. We also excluded patients with acquired CM-I secondary to other causes (e.g. cerebrospinal fluid diversion, CNS space-occupying lesions, intracranial hypertension) and patients with other acquired/developmental skull vault or cervical segmentation anomalies (e.g. craniosynostosis, platybasia, basilar invagination, previous posterior fossa surgery). The remaining 57 living individuals did not have CM-I. They underwent CT scanning for health reasons unrelated to the cranium or developmental abnormalities. The DICOM files generated by the CT scanning were converted into 3D models with the aid of the program Slicer3D [25].

**Figure 2.**
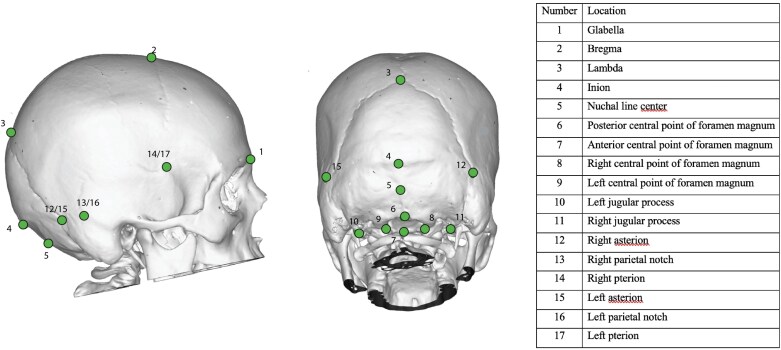
Landmarks used in the present study, shown on a CT-based 3D model of the cranium of living human without CM-I.

We also analysed data from eight fossil hominin crania: (i) Amud 1, (ii) La Chapelle-aux-Saints 1, (iii) La Ferrassie 1, (iv) Singa 1, (v) Skhul IV, (vi) Kabwe 1, (vii) KNM-ER 3733, and (viii) KNM-ER 3883 (Table 1). These fossils were chosen on the basis of the availability of 3D models and the preservation of relevant landmarks. The first three specimens—Amud 1, La Chapelle-aux-Saints 1, and La Ferrassie 1—are generally agreed to be Neanderthals. The next two—Singa 1 and Skhul IV—are widely considered to belong to *H. sapiens*. The taxonomic status of the other three specimens is less straightforward. Many palaeoanthropologists consider E686/Kabwe 1 to be a member of *H. heidelbergensis*, but it has been suggested that the African specimens assigned to *H. heidelbergensis* should be treated as a closely related separate species called *Homo rhodesiensis* [28–30]. Kabwe 1 would be the type specimen of this new species [28–30]. KNM-ER 3733 and KNM-ER 3883 are sometimes assigned to *H. erectus* and sometimes assigned to *H. ergaster*, which is viewed as a close relative of *H. erectus* [31]. We opted to treat  Kabwe 1 as a member of *H. heidelbergensis*, and KNM-ER 3733 and KNM-ER 3883 as members of *H. erectus.* The 3D models of the eight fossil specimens were obtained from collaborators or Morphosource (www.morphosource.com). On each cranial model, the 3D Cartesian coordinates of 17 landmarks were captured using the MorphoDig software package [32]. The locations of the landmarks are shown in Fig. 2. They were chosen to capture cranial shape while also allowing the inclusion of as many fragmentary fossils as possible. According to Bookstein’s [33] criteria, 13 of the landmarks are Type 1, and four are Type 2. Type 1 landmarks have strong homology (e.g. glabella, lambda), while Type 2 landmarks have weak homology (e.g. widest point of foramen).

**Table 1.** Fossil specimens included in the sample used in the present study.

Specimen	Site	Date	Species	References
Amud 1	Amud, Israel	55 ka	*Homo neanderthalensis*	Wood *et al*. [26].
E686/Kabwe 1	Kabwe, Zambia	324-274 ka	*Homo heidelbergensis*	Grün *et al*. [27].
KNM-ER 3733	Koobi Fora, Kenya	*ca.* 1.78-1.65 Ma	*Homo erectus*	Wood *et al*. [26].
KNM-ER 3883	Koobi Fora, Kenya	*ca.* 1.65-1.50 Ma	*Homo erectus*	Wood *et al*. [26].
La Chapelle-aux-Saints 1	La Chapelle-aux-Saints, France	*ca.* 56-47 ka	*Homo neanderthalensis*	Wood *et al*. [26].
La Ferrassie 1	La Ferrassie, France	*ca.* 74-68 ka	*Homo neanderthalensis*	Wood *et al*. [26].
Singa 1	Singa/Sinjah, Sudan	152.5-79.7 ka	*Homo sapiens*	Wood *et al*. [26].
Skhul IV	Skhul, Israel	130-100 ka	*Homo sapiens*	Wood *et al*. [26].

Once we had collected the landmark data, we removed the confounding effects of translation, rotation, and size. To do so, we subjected the dataset to generalized Procrustes analysis (GPA). GPA scales landmark configurations to centroid size and removes translational and rotational effects, which means that it allows specimens to be compared on the basis of true shape [34–36]. The GPA was carried out in Morphologika [37].

Subsequently, we tested for another potenial confounding effect—sexual dimorphism. To do so, we subjected the Procrustes coordinates to principal components analysis (PCA). To reduce noise introduced by PCs that account for little variance, we included only the PCs that account for 5% or more of the total shape variance in further analysis, as per Zelditch *et al*. [35] and Plomp *et al*. [26, 38]. We ran a MANOVA on the retained PCs and compared the cranial shape of female and male living humans. The PCA was performed in R [27] and the MANOVA was performed in SPSS [39]. The MANOVA was insignificant (λ = 0.926, *F* = 1.081, *P* = 0.382), so we continued our analyses with the pooled-sex dataset.

Having controlled for the confounding effects of translation, rotation, and size, and determined that there is negligible sexual dimorphism in the transformed data for living humans, we assigned the individuals in the sample to six operational taxonomic units (OTUs). These were (i) living humans with CM-I, (ii) living humans without CM-I, (iii) fossil *H. sapiens*, (iv) Neanderthals, (v) *H. heidelbergensis*, and (vi) *H. erectus*.

Subsequently, we carried out two sets of analyses. The goal of the first was to test the key assumption of the Archaic *Homo* Introgression Hypothesis, which is that CM-I is associated with significant differences in cranial shape, especially with respect to the basicranium. We began by subjecting the Procrustes coordinates for the two living human OTUs to PCA. Again, we retained only the PCs that accounted for 5% or more of the total shape variance [35]. Next, we subjected the retained PCs to a MANOVA to determine whether or not there were significant differences between the two OTUs. After this, we carried out two analyses to clarify the nature of the shape differences between affected and unaffected individuals. To begin with, we analysed the retained PCs with canonical variates analysis (CVA). CVA maximizes the between-group variance while minimizing the within-group variance [35, 36]. To visualize the shape differences captured by the CVs, we generated a histogram and wireframes. Subsequently, we plotted the retained PCs against each other and used wireframes to identify the major changes in shape along the PCs.

The goal of the second set of analyses was to test the main prediction of the Archaic *Homo* Introgression Hypothesis, which is that the crania of people with CM-I should be more similar in terms of shape to the crania of *H. erectus*, *H. heidelbergenesis*, and *H. neanderthalensis* than are the crania of unaffected people. We began by adding the Procrustes coordinates for the four  fossil OTUs to the Procrustes coordinates for the two living human OTUs. We then ran a PCA on the combined dataset and again reduced noise by excluding PCs that accounted for less than 5% of the total variation. Next, we calculated the Procrustes distances between the living human OTUs and each of the fossil OTUs. After this, we sought to determine whether the fossil specimens differ from unaffected living humans in the same way as living humans with CM-I differ from unaffected living humans. To do this, we performed a CVA on the retained PCs for all the OTUs and generated scatter plots and wireframes. We also plotted the retained PCs against each other and used wireframes to identify changes in shape along the PCs.

The two sets of analyses were carried out with the aid of R [27] and SPSS [39].

## RESULTS

### Comparison of living humans with and without CM-I

The PCA that compared the two living human OTUs yielded seven PCs that met the criterion for inclusion. Collectively, these PCs accounted for 56% of the shape variance.

The MANOVA performed on the seven retained PCs was significant (λ = 0.646, *F* = 7.434, *P* < .001), which indicates that there are differences in the shapes of the crania of individuals with and without CM-I.

**Figure 3.**
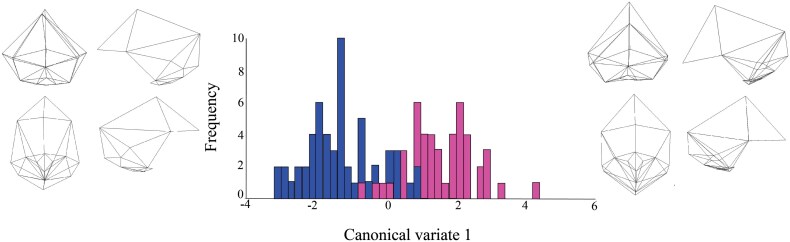
Histogram depicting the distribution of the scores of the two living human OTUs on the single CV yielded by the CVA. Pink bars = individuals with CM-I. Blue bars = individuals without CM-I. The wireframes illustrate the shapes at the ends of the CV. From top left to bottom right, wireframes show neurocranium in posterior, left lateral, inferior, and right lateral orientations.

The CVA yielded a single CV due to the inclusion of two groups. There is relatively little overlap between the two OTUs on this CV (Fig. 3). Individuals with CM-I (pink bars) tend to be positioned more towards the positive end of the CV while those without CM-I (blue bars) tend to be located more towards the negative end. In comparison to individuals without CMI, individuals with CM-I tend to have reduced cranial vault height, reduced occipital height, and reduced occipital breadth. They also tend to have a lower occipital protuberance and a lower asterion. In addition, there are differences in the size and location of the foramen magnum. Specifically, the foramen magnum tends to be smaller and located more anteriorly in individuals with CM-I than in individuals without CM-I. Lastly, there are differences in relation to the positions of the pterion and bregma relative to one another: at the end of the CV that is dominated by individuals without CM-I, the pterion is positioned anterior to bregma, whereas at the end of the CV is dominated by individuals with CM-I, bregma is located anterior to pterion.

**Figure 4.**
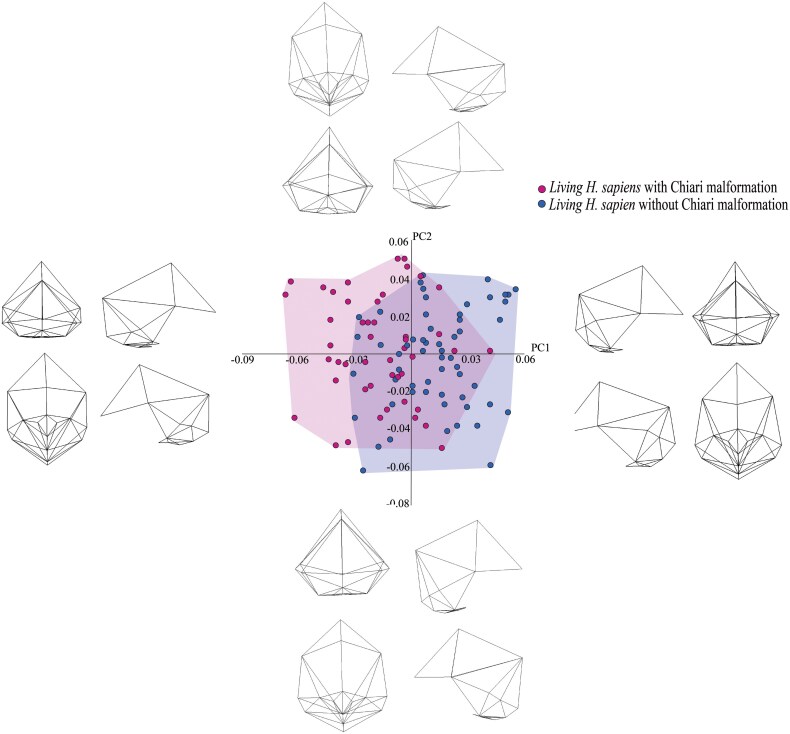
PCA illustrating the shape variation among the living human subsample when PC2 is plotted against PC1. The pink circles are individuals with CM-I; blue circles are unaffected individuals. The wireframes show the shapes at the end of each PC.

Figure 4, which plots PC1 (12% of the variation) against PC2 (11% of the variation), also illuminates the shape differences between the two living human OTUs. There are no obvious differences on PC2, but several are discernible on PC1, the axis explaining the greatest variation in the sample. The morphological differences are largely the same as those identified in the CVA (Fig. 3). Specifically, the main differences between individuals with and without CM-I relate to a flattening of the occipital and caudal location of the lambda and glabella. One difference that is captured by the PC plot but not by the CVA one is that individuals with CM-I tend to have a relatively smaller foramen magnum than individuals without CM-I.

Taken together, the results of the first set of analyses indicate that the crania of living humans with CM-I are significantly different in terms of shape from the crania of living humans without CM-I. They also indicate that the shape differences between living humans with and without CM-I are especially apparent in the basicranium. Thus, the results of the first set of analyses support the key assumption of the Archaic *Homo* Introgression Hypothesis.

### Comparison of living humans with and without CM-I to fossil OTUs

The PCA that included all six OTUs yielded seven PCs that met the criterion for inclusion. Together, these PCs accounted for 57% of the shape variance.

The Procrustes distances between the two living human OTUs and the fossil OTUs are listed in Table 2. The distances show that living humans with CM-I are closer in shape to Neanderthals than living humans without CM-I, while living humans without CM-I are closer in shape to *H. erectus*, *H. heidelbergensis*, and fossil *H. sapiens*.

**Table 2.** Results of analysis in which Procrustes distances were calculated between the two living human OTUs and the other four OTUs.

	Living humans with CM-I	Living humans without CM-I
**Fossil *H. sapiens***	0.0564	0.0493
***H. neanderthalensis***	0.0973	0.1009
***H. heidelbergensis***	0.0679	0.0649
***H. erectus***	0.1421	0.1356

For each pair of values, the underlined one is the smaller of the two distances.

**Figure 5.**
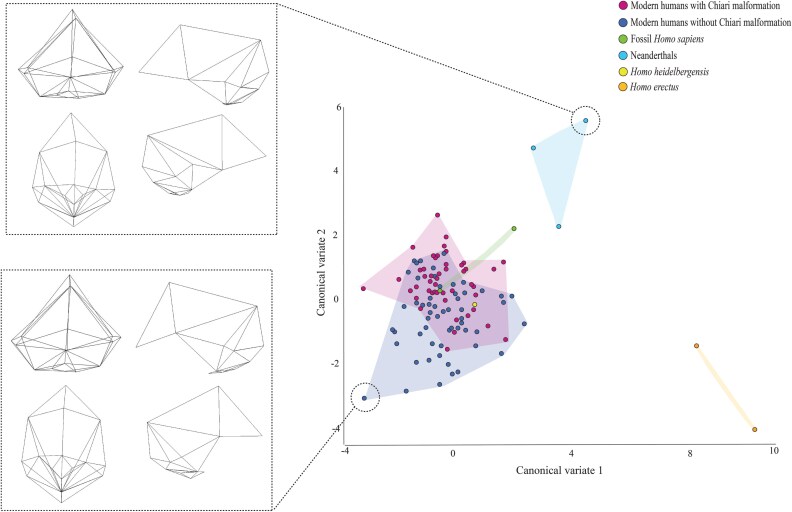
CVA plot depicting the between-group shape variation when CV2 is plotted against CV1. The wireframes illustrate the shape differences between individuals at the positive and negative ends of CV2.

The CVA performed on the retained PCs yielded five CVs, due to the inclusion of six groups. The scatter plot in Fig. 5 shows CV2 (26% of the variation) plotted against CV1 (58% of the variation). None of the other scatter plots generated from the CVs revealed noteworthy patterns, so we will not discuss them.

There are three clusters of specimens in the CVA plot in Fig. 5. One of these clusters consists of the two *H. erectus* specimens. These specimens are located towards the positive end of CV1 and the negative end of CV2. A second cluster is formed by the three Neanderthal specimens. This cluster is located close to halfway along CV1 and at the positive end of CV2. The third cluster is the largest of the three and is positioned towards the negative end of CV1 and the middle of CV2. It comprises the living humans with CM-I, the living humans without CM-I, the two fossil *H. sapiens* specimens, and the *H. heidelbergensis* specimen. Within this cluster, the living humans with CM-I are, in general, located more towards the positive end of CV2 than are the living humans without CM-I. One of the two fossil *H. sapiens* specimens overlaps with both living human OTUs but the other aligns solely with the living humans with CM-I on CV2. The *H. heidelbergensis* specimen is located well within the zone of overlap between the two living human OTUs, close to the centre of CV2.

Because no clear differences between living humans with and without CM-I are discernible on CV1, we will concentrate on the shape changes that occur on CV2, which can be understood with the aid of the wireframes at the top and bottom of Fig. 4. Compared to living humans without CM-I, living humans with CM-I tend to have a less globular cranial vault, more caudally located pterions and lambdas, relatively smaller foramen magnums, and flatter occipital bone, especially posterior to the foramen magnum (i.e. the squamous part). The Neanderthal specimens differ from the living humans without CM-I in the same way, as do the fossil *H. sapiens* specimens.

**Figure 6.**
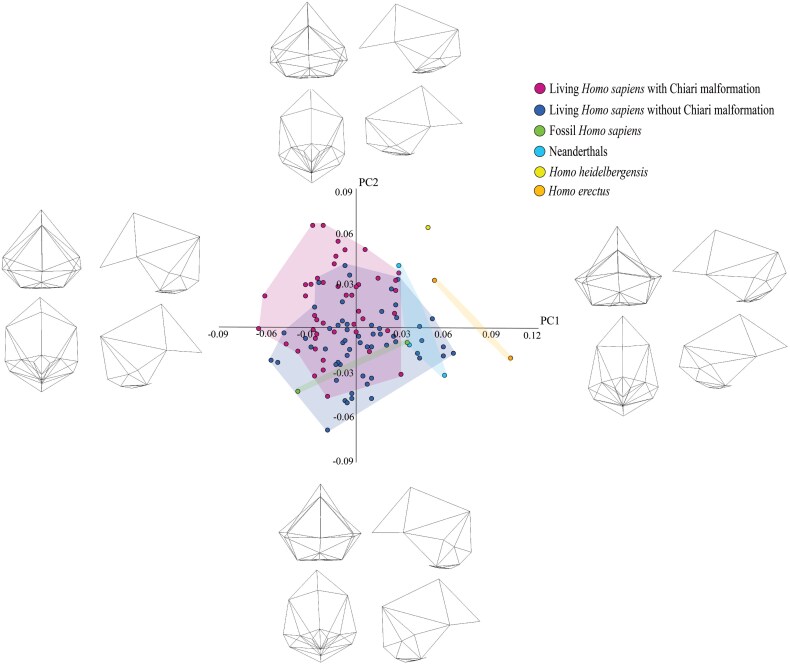
PCA depicting the shape variance within the entire sample when PC2 is plotted against PC1. The wireframes illustrate the shapes at the extreme end of each PC.

Plotting the seven PCs against each yields a complementary picture of the shape differences among the taxa. As with the CV plots, only one of the PC plots yielded a noteworthy pattern: PC1 (12% of the variation) vs. PC2 (10% of the variation). In this plot, which is shown in Fig. 6, there is one main cluster of specimens. This consists of the living humans with and without CM-I, the two fossil *H. sapiens* specimens, the three Neanderthal specimens, and the *H. heidelbergensis* specimen. Within this cluster, the living humans without CM-I overlap more with the fossil *H. sapiens* and Neanderthal specimens than do the living humans with CM-I. The *H. heidelbergensis* specimen overlaps with living humans without CM-I on PC1 and with living humans with CM-I on PC2. *H. erectus* plots more positively on PC1 than the other OTUs but overlaps with all the other OTUs except *H. heidelbergensis* on PC2.

It is clear from the wireframes associated with Fig. 6 that there are no substantive differences between living humans with and without CM-I on PC1. Accordingly, we will concentrate on the shape differences that are discernible on PC2. The most obvious of these relates to the squamous part of the occipital bone. This tends to be relatively short along the sagittal plane in living humans with CM-I and *H. heidelbergensis* compared to living humans without CM-I, *H. erectus*, *H. neanderthalensis*, and fossil *H. sapiens*.

The results of the second set of analyses are inconsistent with the main prediction of the hypothesis. The finding that the crania of living humans with CM-I are more similar to those of *H. neanderthalensis* than are the crania of living humans without CM-I is in line with the prediction. However, the fact that the analyses indicate that living humans without CM-I are closer in shape to *H. heidelbergensis* than living humans with CM-I is not in line with the test prediction. Nor is the fact that the Procrustes distances indicate that living humans without CM-I are closer in shape to *H. erectus* than living humans with CM-I.

## DISCUSSION AND CONCLUSIONS

In the study reported here, we applied 3D shape analysis techniques to models of the crania of living humans with and without CM-I and several fossil hominin crania to evaluate Fernandes *et al*’.s [16] introgression-based hypothesis for CM-I. To recap, Fernandes *et al*. [16] argued that individuals develop CM-I because some of their cranial development-coding genes derive from three archaic *Homo* species—*H. erectus*, *H. heidelbergensis,* and *H. neanderthalensis*. The genes in question, Fernandes *et al*. [16] averred, entered the modern human gene pool via interbreeding events during the Pleistocene.

We conducted two sets of analyses. In the first, we focussed on the living humans in the sample and evaluated the key assumption of Fernandes *et al.*’s [16] hypothesis, which is that CM-I is associated with significant differences in cranial shape, especially with respect to the basicranium. The analyses identified a number of significant differences in shape. The analyses indicated that, compared to individuals without CM-I, individuals with CM-I tend to have reduced cranial vault height; reduced occipital height and width; a more inferiorly located asterion and inion; a more posteriorly located pterion; and a more anteriorly located and smaller foramen magnum. Given that several of these shape differences relate to the basicranium, the results of the first set of analyses are consistent with the hypothesis’ key assumption.

In the second set of analyses, we compared the crania of living humans with and without CM-I to a number of fossil specimens. The goal of this set of analyses was to test the main prediction of the hypothesis, which is that the crania of living humans with CM-I should be closer in shape to those of *H. erectus*, *H. heidelbergensis*, and *H. neanderthalensis* than are the crania of living humans without CM-I. The results of the second set of analyses were not in line with this prediction. They indicated that the crania of living humans with CM-I are more similar to those of *H. neanderthalensis* than are the crania of living humans without CM-I, as predicted. However, they also indicated that living humans without CM-I are closer in shape to *H. erectus* and *H. heidelbergensis* than are living humans with CM-I, which is inconsistent with the prediction.

Overall, then, the results of the study were mixed with regard to the Archaic *Homo* Introgression Hypothesis. They support the idea that the crania of people with CM-I differs significantly in terms of shape from the crania of people without CM-I, especially in the basicranium. However, they do not support the idea that individuals develop CM-I because some of their cranial development-coding genes derive from *H. erectus*, *H. heidelbergensis*, and *H. neanderthalensis* as a result of interbreeding.

The simplest explanation for the results we obtained would seem to be that the Archaic *Homo* Introgression Hypothesis is too broad with respect to the species from which the relevant genes were derived. Rather than the genes being traceable to *H. erectus*, *H. heidelbergensis*, and *H. neanderthalensis*, our results are consistent with them being traceable just to *H. neanderthalensis*. The introgressed genes being derived from one or more interbreeding events between *H. sapiens* and *H. neanderthalensis* would explain why in the second set of analyses we found that the crania of living humans with CM-I are more similar to those of *H. neanderthalensis* than are the crania of living humans without CM-I but did not obtain comparable results when we compared the two living human taxa to *H. erectus* and *H. heidelbergensis*. The obvious name for this revised version of the hypothesis is the ‘Neanderthal Introgression Hypothesis’.

Another possible explanation for why our analyses did not support the main prediction of Archaic *Homo* Introgression Hypothesis is that *H. erectus* and *H. heidelbergensis* were represented by so few specimens in our study. To reiterate, we were only able to include one specimen of *H. heidelbergensis* (Kabwe 1) and two specimens of *H. erectus* (KNM-ER3733 and KNM-ER3883). It is undoubtedly the case, then, that a small sample size is a concern with regard to these species. And this concern is magnified when the ranges of variation of the two living human OTUs shown in Figs 5 and 6 are contemplated. If the ranges of variation of *H. erectus* and *H heidelbergenesis* were similar to those of the two living human OTUs, it is not hard to imagine larger samples of the two fossil species being more similar to living humans with CM-I than to living humans without CM-I. Given this, in the next phase of this project, we will try to obtain additional 3D models of fossil specimens assigned to *H. erectus* and *H heidelbergenesis* (and the other fossil taxa included in the sample) and re-run the second set of analyses.

Several other avenues for future research suggest themselves. One of these concerns is the prevalence of CM-I in different regions of the world. The revised version of the hypothesis—i.e. the Neanderthal Introgression Hypothesis—predicts that the prevalence of CM-I should be markedly higher in non-African populations than in African ones. The reason for this is that the percentage of DNA that can be traced to interbreeding with Neanderthals is much lower in living Africans than it is in non-Africans. Recent studies suggest that some African populations carry around 0.3–1.5% Neanderthal DNA, whereas for European and Asian populations the equivalent figure is 1–2.3% [40, 41]. If the Neanderthal Introgression Hypothesis is correct, an obvious implication of the difference in Neanderthal DNA between Africans and non-Africans is that CM-I should be much less prevalent in Africa than it is in Europe and Asia. Currently, it is not possible to test this prediction. CM-I is known to occur among populations of African ancestry [42–44], but there have been far too few studies in Africa to be able to compare the African prevalence rate to the equivalent rates for Europe and Asia with confidence. Importantly, changing this situation would be not only interesting with respect to testing the Neanderthal introgression explanation for CM-I, but would also be useful for improving the well-being of many individuals living in Africa, since it seems likely that CM-I has been underdiagnosed on the continent due to financial constraints.

Another potential avenue for future research is to expand the sample of living humans with CM-I. The individuals with CM-I whose CT scans were used in the present study were a self-selected group and limited to those patients undergoing hospital investigation for their symptoms under a tertiary neurosurgical service. However, a number of studies suggest that a substantial percentage (perhaps as much as 30%) of patients with CM-I can be clinically asymptomatic (e.g. [45, 46],). Thus, in a future study, it would be very useful to include data on a wider range of people with CM-I, including individuals who are asymptomatic and mildly symptomatic.

This study and others have shown that there are differences in cranial shape between adult individuals with and without CM-I. An important next question is, when in ontogeny do the differences emerge? It would also be helpful to know whether the differences develop in tandem or sequentially. It seems likely that these questions could be answered with an approach similar to the one we utilized in the first set of analyses reported here, i.e. by applying 3D geometric morphometrics to digital models derived from CT scans of a sample of individuals of different age, some of whom have CM-I and some of whom do not.

A further possibility for future research is unravelling the relationship between brain size and shape and the size and shape of the braincase in humans with CM-I. Both introgression hypotheses assume that there is a mismatch between the size and shape of the brain and that of the braincase in people with CM-I. However, it is unclear whether/how the size and shape of the brain and the braincase align in such a way as to cause CM-I. A number of studies, including the current one, have identified differences in the shape and size of both the brain and braincase in humans with CM-I, but we have yet to study their 3D shapes in tandem to investigate exactly where the mismatch occurs and how the shape variation of both elements influences the malformation. Thus, it would be useful to directly compare the brains and braincase in a sample of humans with CM-I. Again, this could be accomplished with the techniques employed in the study reported here. Specifically, 3D models of brains and braincases could be generated from CT scans of individuals with and without CM-I, and then 3D geometric morphometric techniques could be used to quantify the relationship between landmarks on each brain-braincase pair of 3D models.

As we noted in the Introduction, clinical studies have identified several potential aetiologies for the small occipital bone associated with CM-I but none of them has been found capable of explaining all cases of the condition. This suggests not only that we should be prepared for the possibility that introgressed genes may be able to explain only some cases of CM-I but also that it would be sensible to investigate whether there are differences in cranial shape among individuals with CM-I that correlate with the different proposed aetiologies. The combination of CT scans and 3D geometric morphometrics used in the present study should be able to shed light on this issue too.

The final point to make here is that the present study adds to our understanding of CM-I regardless of its implications for the idea that the condition involves introgressed genes. Prior to this study, only three cranial traits had been consistently identified as being associated with CM-I: (i) a relatively short posterior fossa [47–50]; (ii) a relatively short clivus [51–55]; and (iii) an anteriorly–posteriorly shorter foramen magnum [54, 55]. The results of our first set of analyses add several traits to the list that, to the best of our knowledge, have not been identified before, including reduced cranial vault height; a more inferiorly located asterion and inion; a more posteriorly located pterion; and a more anteriorly located foramen magnum. It seems likely that this is due to the fact that the present study is the first to use 3D geometric morphometric methods to investigate human cranial shape in relation to CM-I. Given this, it would seem sensible for more researchers interested in CM-I to familiarize themselves with 3D Geometric Morphometrics. The methods would seem to have the potential to help us develop a deeper understanding of the aetiology and pathogenesis of CMs, which could in turn strengthen the diagnosis and treatment of the condition.
